# Olecranon Osteotomy by a Gigli Saw versus Chevron’s Osteotomy for Exposure of Intra-articular Distal Humerus: A Comparative Study

**DOI:** 10.5704/MOJ.2211.011

**Published:** 2022-11

**Authors:** RR Butala, PD Samant, S Mehra

**Affiliations:** Department of Orthopaedics, Padmashree Dr DY Patil University, Navi Mumbai, India

**Keywords:** Gigli saw, olecranon osteotomy, chevron osteotomy, distal humeral fracture, osteotome

## Abstract

**Introduction:**

Olecranon osteotomy is employed for the fixation of intraarticular distal humeral fractures. We conducted a prospective, randomised study comparing Chevron’s osteotomy with olecranon osteotomy by a Gigli saw for exposure of the intraarticular distal humerus in terms of functional outcome and intra-operative ease of the surgery.

**Materials and methods:**

Thirty patients with skeletally mature AO/OTA type 13- B and 13-C distal humerus fractures were randomly allocated to Chevron’s or Gigli saw groups. Each group consisted of a total of 15 patients. Both the groups were assessed on post-operative parameters including arm, shoulder or hand pain, ability to perform certain routine activities, tingling sensations and pain while sleeping.

**Results:**

In the Gigli saw group, 12 patients had no gross limitation of activity and 13 were able to perform moderate activities with ease. Similar results were observed in the Chevron’s group. The mean difference between the two groups in Oxford Score was 0.60, within the 95% confidence interval and in line with QuickDASH-11 Score.

**Conclusion:**

Chevron’s technique offers stability and better healing, providing a larger surface area for bone union. However, it is challenging and time-consuming. Also, literature suggests that the Gigli saw has multiple benefits, saves time and effort, and heals by switching blood supply from centrifugal to centripetal post-operatively. Our study suggests that both Chevron’s technique and the use of the Gigli saw are effective in distal humeral intra-articular fractures as assessed by multiple parameters. Hence both techniques can be equally used depending on the surgeon’s preference.

## Introduction

Olecranon osteotomy is a standard technique employed for open reduction and internal fixation of intra-articular distal humeral fractures^1^. This technique has better joint visualisation when compared to non-osteotomy techniques^2-3^. Although many different types of techniques have been used^4^, the Chevron’s technique is preferred because it offers stability as the post-osteotomy olecranon portion is fitted into the wedge of the proximal ulna cut. It also provides a large surface area of the bone, which aids in healing. However, the Chevron’s technique is difficult to perform, and it has limitations as it is time-consuming, and skill is required to use the motorised saw^5^. In addition, there is also the possibility of skewing when using the saw which results in change in a change in direction and shape of the osteotomy. Thus, Chevron’s osteotomy is technically difficult despite being effective.

A less popular technique can be used as an alternative by using a Gigli saw to perform the procedure. This technique is less time-consuming and at the same time easy to perform as it is done manually, compared to the Chevron’s osteotomy^5^. This study was performed to compare the healing potential and intra-operative problems faced during the two types of osteotomies, and their post-operative outcomes of pain, joint stiffness, and range of motion.

## Materials and Methods

This study was a prospective, randomised controlled study comparing Chevron’s osteotomy to an olecranon osteotomy by a Gigli saw for the fixation of intraarticular distal humeral fractures. It was conducted from December 2018 to August 2020, at the Department of Orthopaedics of a tertiary care teaching hospital in Navi Mumbai.

The inclusion criteria were patients with the distal end humerus fracture with an intra-articular extension; pain and limited range of motion in the affected region; and a signed informed consent to participate in the study and comply with follow up schedules.

The exclusion criteria were a post-surgical history of olecranon fractures; arthritis of elbow joint; pain of elbow region with a secondary aetiology; any surgical intervention in the affected region in the last 12 months; any post-surgery/tumour history; participation in another study in the last one month; post-operative surgical site infection; refusal to participate in the study and refusal to give an informed written consent.

Eligible patients were enrolled in the study after obtaining a study-specific informed written consent. The study protocol was approved by the institutional ethics committee (IEC) of the institute and was conducted along the good clinical practice guidelines and the Declaration of Helsinki.

Fifty-three patients with skeletally mature AO/OTA type 13-B and 13-C distal humerus fractures were assessed for eligibility, and 23 were excluded following the inclusion and exclusion criteria. The remaining thirty were divided into two groups with 15 adult participants in each study group. Detailed history was taken from every participant. The patients were operated on, with either the Gigli saw or the Chevron’s method, using a computer-generated randomisation schedule of three blocks, with ten patients per block.

At surgery, the patient was laid in the lateral position with the arm resting over the armrest. Using the standard posterior approach, an incision was made, penetrating through subcutaneous tissue, and the ulnar nerve is identified, and dissected. Capsular dissection was done, making an entry point from both medial and lateral side of the olecranon. In each case the osteotomy was fixed by tension band wiring.

**Fig. 1.**
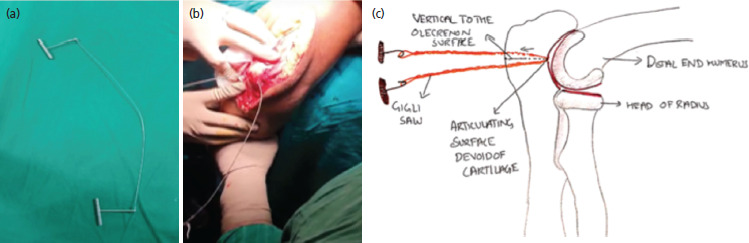
(a) An image of the instrument used in Gigli saw; (b) An intra-operative image of olecranon osteotomy with Gigli saw in a 54-year-old female, a post-traumatic case of right sided intra-articular distal humerus fracture closed with no distal neurovascular deficit; (c) An illustrative image depicting the to and fro motion of Gigli saw going from inside out vertically to the surface of olecranon.

**Fig. 2.**
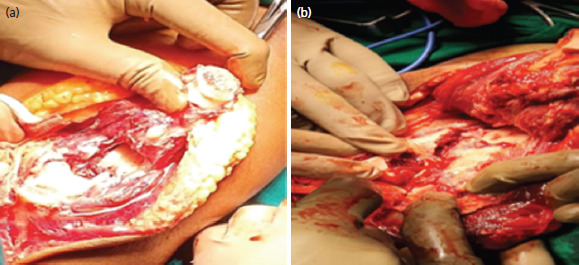
(a) Intra-operative exposure of distal humerus post olecranon osteotomy with Gigli saw in a 54-year-old female, a post-traumatic case of right sided intra-articular distal humerus fracture closed with no distal neurovascular deficit; (b) Image showing humerus exposure being done.

**Fig. 3.**
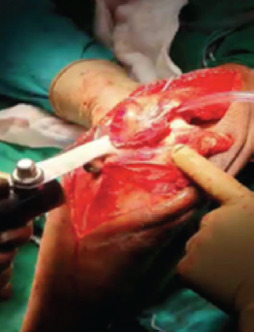
An intra-operative image of olecranon osteotomy with chevron’s technique in a 55-year-old male, a post-traumatic case of right sided intra-articular distal humerus fracture closed with no distal neurovascular deficit.

A Gigli saw (Fig. 1a) was placed vertically and passed anterior to the olecranon between the ulno-humoral joint, from lateral to medial, using a long- curved haemostatic artery forceps (Fig. 1b). It was used to create an osteotomy through the bare area of the sigmoid notch, approximately two cm distal to the tip of olecranon, using gentle to and fro movement of the hand, moving from inside out (Fig. 1c). The proximal olecranon fragment, and extensor mechanism were retracted en bloc proximally, to expose the articular surface. The joint was visualised, and osteotomy was positioned to enter the joint where the semilunar notch was devoid of cartilage^6^. Fig. 2 demonstrates an olecranon osteotomy using the Gigli saw in a 54-year-old female. Similarly, in Chevron’s osteotomy, a motorised saw was used from outside-in and the last part near the cartilage was broken with the help of an osteotome. The fracture fragments were easily identified and fixed with bi-pillar plating; the osteotomy fragments were approximated again and fixed with k-wire and tension This was band wiring. Fig. 3 shows an intra-operative image of chevron’s technique performed on a 55-year-old male.

In each case, immediate post-operatively sterile dressing was applied. Patients were made to perform active finger movements and passive elbow range of motion exercise from post-operative day 1. This was followed by active range of motion and shoulder range of motion exercises from postoperative day 2. All patients underwent a dressing check on post-operative day 2 as well as day 5. Patients were discharged on post-operative day 5 and were followed up on post-operative day 14 for suture removal. The patients were asked to attend follow ups after three weeks, one month, three months and six months post-surgery. During this time all patients were subjected to QuickDASH, a short version of the Disabilities of the Arm, Shoulder and Hand questionnaire, and the Oxford Elbow Scoring System, to assess and compare the post-operative outcomes in both techniques^7-8^.

**Fig. 4.**
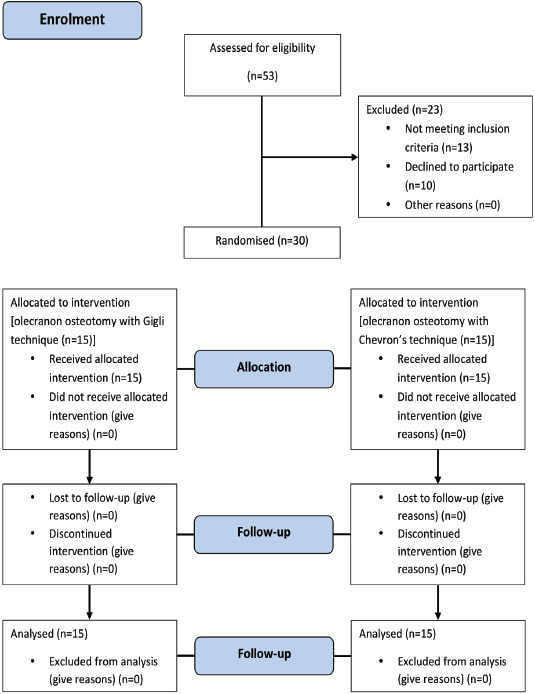
Consort 2010 flow diagram.

In accordance with the GCP guidelines, informed consent was obtained from each participant before any study related procedures. Statistical analysis was done using the windows-based program SPSS (Statistical Package for the Social Sciences) version 17. The age, duration of surgery, blood loss and incision length were compared between the two groups using an independent sample t-test; whereas the ranking data scores for the Oxford and QuickDASH-11 for individual items and total scores were compared between the two groups using the non- parametric Mann-Whitney ‘U’ test. Discrete data for the patients in categories for gender and side of surgery were analysed using the chi-square test. All analyses were done using the two-sided tests at alpha 0.05 (95% confidence level). Fig. 4 presents the patient flow as per the CONSORT 2010 flow diagram^8^.

## Results

The severity of symptoms was measured by several parameters including the range of motion, pain, and limitation of any activity using the Oxford and QuickDASH Scores. The assessment of functions was done postoperatively, at three weeks, one month, three months and six months post-surgery. In the Gigli saw group, thirteen patients had a good range of motion after the procedure. Two patients experienced post-operative pain whereas three patients had experienced mild symptoms of arm and shoulder pain. Furthermore, tingling sensation persisted in arm, hand, or shoulder of two patients which recovered, with activity after six weeks. No sleep issues were observed due to postoperative pain. Of the 15 patients in the Chevron’s osteotomy group, 12 had no gross limitation of activity as a result and 13 patients could perform moderate activities with ease.

**Fig. 5.**
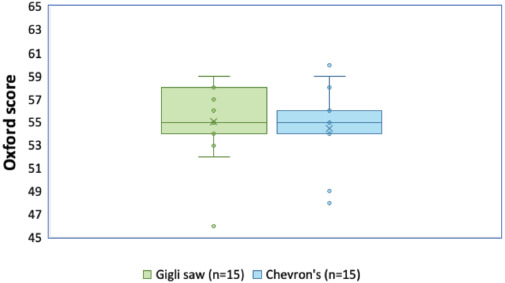
Oxford Scores (median and quartiles) with two techniques.

**Fig. 6.**
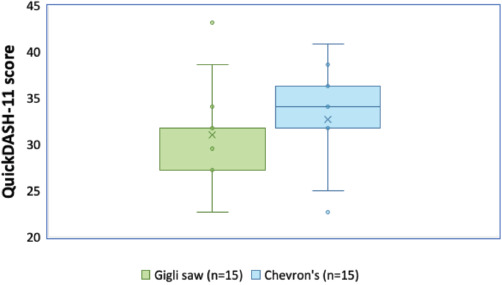
QuickDASH-11 Scores (median and quartiles) with two techniques.

The demography and profile of the patients with the mean surgery duration are shown in Table I. Both groups had 15 patients. There were ten males and five females in the Gigli saw group, and eight males and seven females in Chevron’s osteotomy group. The mean age of the Gigli saw group was 45.20 years whereas that of the Chevron’s osteotomy group was 44.67 years. In the Gigli saw group, the side most operated was right side and it was the left side in the Chevron’s group. The mean difference in Oxford and QuickDASH Scores of the two groups was 0.60 (p-value = 0.834) and 1.67 (p-value = 0.177) (as shown in Table II and Table III) which indicated that there was no significant difference between the two techniques. Furthermore, the difference in the mean score for individual parameters (this mean score was the mean of scores measured at all time points) between Oxford and QuickDASH was within 95% confidence interval as shown in Table II and Table III respectively, and also represented in Fig. 5 and Fig. 6 with median and quartile.

**Table I:** Demography and profile of patients in two groups

	Gigli saw (n=15)		Chevron’s (n=15)		p
	Mean	SD	Mean	SD	t-test
Age (yrs.)	45.20	1.33	44.67	1.95	0.823
Blood loss (ml)	347.60	9.72	3393.35	6.69	<0.0001
Incision length (cm)	15.05	1.84	15.30	1.30	<0.0001
Surgery duration (min.)	80.60	3.73	85.00	4.66	<0.0001
	**No.**	**%**	**No.**	**%**	**χ2-test**
**Gender**					
Male	10	66.7%	8	53.3%	0.456
Female	5	33.3%	7	46.7%	
**Operated Side**					
Left	4	26.7%	9	60.0%	0.065
Right	11	73.3%	6	40.0%	

**Table II:** Scores for Oxford items in two groups

	Gigli Saw (n=15)			Chevron’s (n=15)			Z	p
	Mean	Median	(25 – 75 percentiles)	Mean	Median	(25 – 75 percentiles)		
Interfered with your usual work	4.60	5	(5-5)	4.47	5	(3-5)	-0.424	0.671
Usual pain	4.67	5	(4-5)	4.60	5	(4-5)	-0.362	0.718
Pain interfered with sleeping	4.47	5	(3-5)	4.47	5	(3-5)	0.000	1.000
Difficulty lifting things	4.60	5	(5-5)	4.47	5	(3-5)	-0.424	0.671
Difficulty carrying bags of shopping	4.60	5	(5-5)	4.47	5	(3-5)	-0.424	0.671
Difficulty washing yourself	4.47	5	(3-5)	4.60	5	(5-5)	-0.424	0.671
Difficulty dressing yourself	4.60	5	(5-5)	4.47	5	(3-5)	-0.424	0.671
Controlling your life	4.73	5	(5-5)	4.73	5	(5-5)	0.000	1.000
On your mind	4.47	5	(3-5)	4.47	5	(3-5)	0.000	1.000
Pain in bed at night	4.60	5	(5-5)	4.47	5	(3-5)	-0.424	0.671
Limited ability to take part in leisure activities	4.47	5	(3-5)	4.47	5	(3-5)	0.000	1.000
Worst pain	4.80	5	(5-5)	4.80	5	(5-5)	0.000	1.000
Total Oxford score	55.07	55	(54-58)	54.47	55	(54-56)	-0.210	0.834

* Higher scores are better (minimum dysfunction) Oxford scores: Score 0 to 19 – Severe elbow arthritis. Score 20 to 29 – May indicate moderate to severe elbow arthritis. Score 30 to 39 - May indicate mild to moderate elbow arthritis. Score 40 to 48- May indicate satisfactory joint function

**Table III:** Scores for QuickDASH items in two groups

	Gigli Saw (n=15)			Chevron’s (n=15)			Z	p
	Mean	Median	(25 – 75 percentiles)	Mean	Median	(25 – 75 percentiles)		
Regular daily activities	1.40	1	(1-1)	1.40	1	(1-1)	0.000	1.000
Normal social activities	1.40	1	(1-1)	1.53	1	(1-3)	-0.424	0.671
Tingling	1.20	1	(1-1)	1.33	1	(1-2)	-0.812	0.417
Wash your back	1.20	1	(1-1)	1.20	1	(1-1)	0.000	1.000
Do heavy household chores	1.33	1	(1-1)	1.47	1	(1-2)	-0.449	0.653
Difficulty have you had sleeping	1.13	1	(1-1)	1.20	1	(1-1)	-0.482	0.630
Open a tight or new jar	1.40	1	(1-1)	1.40	1	(1-1)	0.000	1.000
Do heavy household chores	1.40	1	(1-1)	1.53	1	(1-3)	-0.424	0.671
Use a knife to cut food	1.27	1	(1-1)	1.40	1	(1-1)	-0.482	0.630
Recreational activities	1.53	1	(1-3)	1.40	1	(1-1)	-0.424	0.671
Arm, shoulder, or hand pain	1.40	1	(1-1)	1.53	1	(1-3)	-0.424	0.671
Total QuickDASH score	31.06	31.82	(27.27-31.82)	32.73	34.09	(31.82-36.36)	-1.350	0.177

* Lower scores are better (minimum dysfunction)

There were fewer chances of associated injuries to the trochlea or the cartilage by the Gigli saw osteotomy of the olecranon as it was done manually and done inside out. Restriction of movement as well as joint stiffness were not seen frequently.

## Discussion

An olecranon osteotomy provides an excellent view of the articular surface in complex distal humeral fractures compared with triceps-sparing or triceps-splitting approaches. However, the commonly used Chevron’s osteotomy can be technically difficult and needs fixation^5^. An olecranon osteotomy using Gigli saw is technically easier.

In this study, patients were randomised into two groups to receive either the Chevron’s osteotomy or the Gigli saw for the olecranon osteotomy; followed by a statistical comparison between the outcomes and follow up scores. Osteotomy union was achieved in all cases with both techniques, and the anatomical and functional outcome scores (QuickDASH, Oxford Elbow Scoring System and VR-12) showed good results in all the patients.

Table I shows the demography and profile of the patients. Table II and Table III show comparison between the mean Oxford and QuickDASH-11 Scores of the two groups. It was observed that the mean difference between the two groups in the Oxford Score was 0.60. It can be said with 95% confidence that there was no difference in the outcome of the two techniques. Similarly, the mean difference in QuickDASH-11 Score was also within the 95% confidence interval. Both these scores indicated that there was no significant difference in the outcomes of the Gigli saw and Chevron techniques.

Moreover, as the Gigli saw is moved in an inside out fashion, the cartilaginous damage is very minimal. The Chevron technique utilises an osteotome to crack, rather than remove the cartilage itself^9^. In this case, a possibility of the creation of a large cartilaginous flap exists when the wedge is opened forcefully leading to cartilage damage^10-11^. However, because the Gigli saw technique ensures proper anatomic reduction, there will be no chance of articular step-off, and the shape of the olecranon is preserved.

**Fig. 7.**
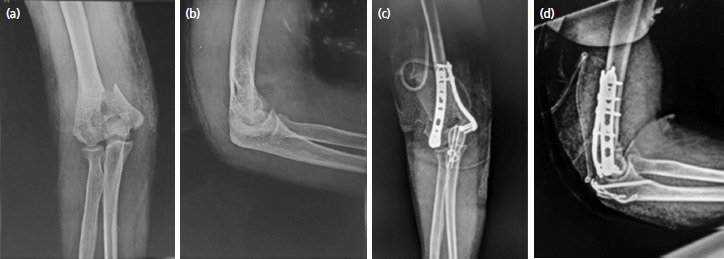
(a) Pre-operative radiograph of elbow after Gigli saw in AP and (b) lateral view of a 54-year-old female, a post-traumatic case of right sided distal humerus intra-articular fracture closed with no distal neurovascular deficit; (c) Post-operative radiograph of elbow in AP and (d) lateral view of a 54-year-old female, a post-traumatic case of right sided distal humerus intra-articular fracture closed with no distal neurovascular deficit.

**Fig. 8.**
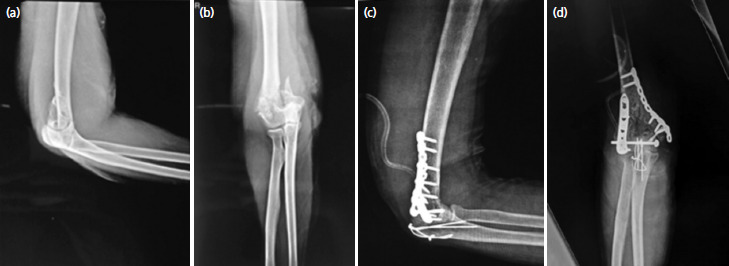
(a) Pre-operative radiograph of elbow after chevron’s osteotomy in AP and (b) lateral view of a 55-year-old male, a post-traumatic case of right sided distal humerus intra-articular fracture closed with no distal neurovascular deficit; Post-operative radiograph of elbow in (c) AP and (d) lateral view of the same patient.

By contrast, despite having minimal cartilage loss, the Chevron technique has more chance of articular incongruence due to nonanatomic reduction, and achieving anatomic reduction is more time-consuming and challenging. The Oxford Scores (refer Table II) and QuickDASH Scores (refer Table III) showed that there was no significant difference between the two techniques when follow up was done. The success of both the techniques were assessed on parameters like pain, pain during sleep, doing heavy household chores and performing other activities. The mean difference between the two on all parameters was within the 95% confidence interval demonstrating similar outcomes. Furthermore, the radiograph of a patient after osteotomy with Gigli saw (as shown in Fig. 7) was similar to the radiograph of patient after Chevron’s osteotomy (as shown in Fig. 8).

A final note is that the Gigli saw technique creates a flat surface osteotomy that has a smaller surface area and a smaller olecranon fragment than the Chevron’s technique^5^. There is a theoretical chance of this increasing the likelihood of non-union. To mitigate this, the Gigli saw is pulled distally as it is pulled from inside-out fashion. This creates a broad, oblique cut with a large olecranon fragment and a bleeding surface at metaphysis region^12^.

After an olecranon osteotomy is performed, the force with which the blood flows, changes from centrifugal to centripetal which leads to a constant blood supply predominantly to the periosteum^13^. The Gigli saw also causes less periosteum damage and less trauma than a conventional saw^13-14^. Also, utmost care needs to be taken while using a power saw as the metal and bone debris generated could further accelerate wear and tear^13^. Gigli saw overcomes these shortcomings. Multiple studies have also demonstrated that electric saws generate noise and vibrations that lead to tingling and numbness of fingers among orthopaedic surgeons^15^. Moreover, Gigli saw employs a small space is less than 500μm and smooth cut which heals faster due to more contact between the surfaces^16-18^. Additionally, during COVID-19 pandemic, it is recommended to consider using Gigli saw as a precautionary measure^19^.

In this series, our sample size was small, and the follow-up period was short, but within this period, all osteotomy sites healed and there were no complications related to the two techniques of the osteotomy of the olecranon.

Osteotomy with the Gigli saw was smooth, uniform and with no periosteal damage; there was adequate exposure for fixing. It has been observed that the healing of the olecranon osteotomy is better and more economical with the Gigli saw, producing minimal injuries compared to other methods. There are fewer chances of iatrogenic fractures, cartilage and trochlear injuries as the osteotomy is performed inwards to outwards. Overall, it has a superior outcome without any complications.

However, this study had certain limitations as the calculation of mean healing time in each group was not performed in this study and the sample size was not adequate for generalisation of the results. Also, exact intra-operative times for the osteotomies was not taken into investigation. Therefore, the above limitation can be taken into consideration in future studies with a larger sample size.

## Conclusion

In conclusion, this study effectively compares Chevron’s osteotomy and olecranon osteotomy by a Gigli saw for exposure of intraarticular distal humerus in terms of functional outcome. The results demonstrate no significant difference between the two techniques in terms of the outcome of functional scores. Both the techniques could be used depending on the intra-operative ease of the surgeon. Based on our study, we suggest that osteotomy of olecranon can be carried out with Gigli saw followed by fixation with tension band wiring. Hence, we propose that this approach can be employed as one of the techniques during exposure of distal humerus fractures.
